# Biased Gene Fractionation and Dominant Gene Expression among the Subgenomes of *Brassica rapa*

**DOI:** 10.1371/journal.pone.0036442

**Published:** 2012-05-02

**Authors:** Feng Cheng, Jian Wu, Lu Fang, Silong Sun, Bo Liu, Ke Lin, Guusje Bonnema, Xiaowu Wang

**Affiliations:** 1 Institute of Vegetables and Flowers, Chinese Academy of Agricultural Sciences, Beijing, China; 2 Laboratory of Plant Breeding, Wageningen, The Netherlands; Michigan State University, United States of America

## Abstract

Polyploidization, both ancient and recent, is frequent among plants. A “two-step theory" was proposed to explain the meso-triplication of the Brassica “A" genome: *Brassica rapa*. By accurately partitioning of this genome, we observed that genes in the less fractioned subgenome (LF) were dominantly expressed over the genes in more fractioned subgenomes (MFs: MF1 and MF2), while the genes in MF1 were slightly dominantly expressed over the genes in MF2. The results indicated that the dominantly expressed genes tended to be resistant against gene fractionation. By re-sequencing two *B. rapa* accessions: a vegetable turnip (VT117) and a Rapid Cycling line (L144), we found that genes in LF had less non-synonymous or frameshift mutations than genes in MFs; however mutation rates were not significantly different between MF1 and MF2. The differences in gene expression patterns and on-going gene death among the three subgenomes suggest that “two-step" genome triplication and differential subgenome methylation played important roles in the genome evolution of *B. rapa*.

## Introduction

Genome polyploidization is widespread in all plants, with consequences apparent in the genomes of important crop species [1]. Genome duplication not only provided abundant genetic materials for the evolution of gene family expansion or the foundation of new genes, but might also have produced bulk genetic variations supporting the evolution of plants that are better adapted to diversified environments [2], [3]. The traces of ancient whole genome duplications can be detected by comparative genome analysis. A run of syntenic gene pairs covering approximately the entire genome, constitutes direct evidence for one ancient whole genome duplication [4], [5].

After genome duplication, subgenomes that co-exist in a nucleus differentiate. This differentiation can be observed from comparisons between the two subgenomes: they may differ in both gene density and the level of gene expression [5], [6]. One subgenome is prone to retain more genes, while the other one loses more genes. This phenomenon is referred to as fractionation bias. This bias in gene densities between subgenomes has been observed in *Arabidopsis thaliana* and maize [6], [7], [8], and may be a common feature in species with ancient polyploid genomes [9]. In addition to biased gene fractionation, genes from different subgenomes also show expression differences. The subgenome that harbors significantly more genes also expresses to greater RNA levels than the more fractionated subgenome. This phenomenon—called genome dominance—has been observed in several genomes with recent allotetraploidization, such as the allotetraploid of *A. thaliana* and *A. arenosa* [10], the natural allotetraploid *Tragopogon miscellus* [11], as well as an allotetraploid cotton species [12]. Furthermore, in the autotetraploid species maize, the dominant gene expression pattern was also clearly detected, even though the polyploidization occurred about 12 million years ago [5].

For the maize genome, a model to illustrate the subgenome dominance effect has been proposed [5]. This model predicts that the overall rate of gene deletions in the two subgenomes of maize is the same. However, maize genotypes with deletions in the higher expressed duplicate copy of the syntenic genes were removed from the population by purifying selection, while genotypes with deletions in the lower expressed copy were more likely to be under near-neutral selection, as their fitness may not be reduced. Subsequently, the difference in selection pressure over the two subgenomes resulted in the observed biased gene fractionation.

**Figure 1:**
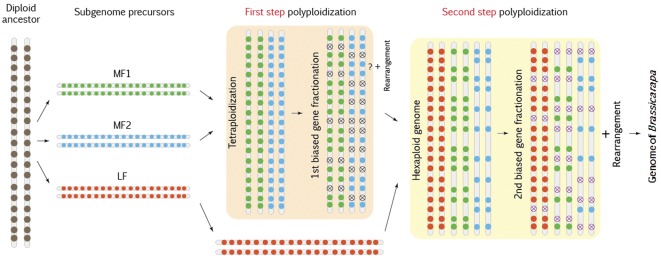
Flow chart of the “two-step theory" to explain the genome triplication that occurred in the early stages of the origin of *B. rapa* species. Circles denote genes and circles with crosses indicate genes that are not detectable. Red circles are genes in subgenome LF, blue and green circles are genes in subgenomes MF1 and MF2, respectively.

The mesopolyploid crop species *Brassica rapa*, a member of the Brassicaceae family, has three subgenomes in its nucleus, representing a genome triplication event that preceded the origin of the diploid Brassica species, *B. rapa*, *B. oleracea*, and *B. nigra* [13]. *B. rapa* is a good model for studying genome polyploidization because the triplicated genome is old enough to be fractionated, but young enough such that most genes are clearly identifiable in the out-group, *A. thaliana* [14], [15]. Segments of the three subgenomes in *B. rapa* are well distinguished; therefore, the difference in gene loss among these subgenomes can be identified unambiguously. We proposed a “two-step theory" to explain the genome triplication events that occurred in *B. rapa* (Figure 1) [13]. In the present study, we found further evidence for differential subgenome evolution, suggesting that differential methylation may have played an important role in the genome evolution of *B. rapa*.

## Results

### Reconstruction and definition of the three subgenomes in *B. rapa*

The genomes of species from Brassicaceae comprise 24 genomic blocks (A-X, also called the ancestral karyotypes, AK) [16], [17], which can be observed in the recently sequenced genomes of *A. lyrata*, *Thellungiella parvula*, and *B. rapa* [13], [18], [19], as well asthe model plant *A. thaliana* and others [20]. . These genomes were all generated from polypoidization, followed by rearrangement of the 24 AK blocks. Many species from the genus Brassiceae have several copies of the AK blocks in their genomes, and it is believed that an ancestral karyotype of Brassiceae (AKBr) existed that contained only one copy of the AK blocks. The genome polyploidization of AKBr gave birth to the polyploid ancestor of the *Brassica* species. Subsequent chromosomal rearrangements, and breaking and fusion of the polyploid ancestor produced the chromosomes of many species in *Brassica*, as exemplified by *B. rapa* with its 10 chromosomes [13], [21]. Given the well annotated *A. thaliana* genome and the few million years of divergence time between *B. rapa* and *A. thaliana*, the genome of *A. thaliana*, which contains only one copy of the AK blocks, was chosen as a representative of AKBr in this study, from which the three copies of AK blocks in *B. rapa* originated.

**Figure 2:**
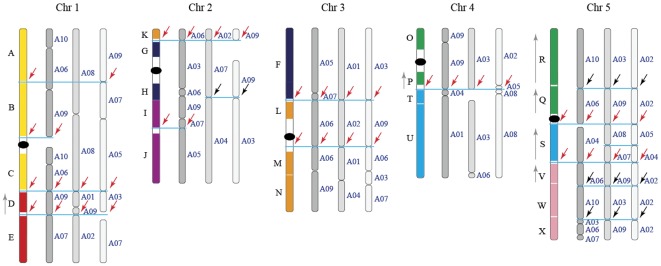
Construction of the three subgenomes in *B. rapa*. Subgenomes are displayed along the five chromosomes of *A. thaliana*, with 24 AK blocks painted in different colors, as reported elsewhere [16]. The deep grey chromosome segments denote subgenome LF, while grey and light grey segments denote MF1 and MF2, respectively. The present-day *B. rapa* chromosomes, such as “A10" from which any segment originates, is labeled to its right. Vertical grey arrows to the left side of block names indicate inverted directions. Horizontal blue lines together with the red arrows indicate identical break regions shared by subgenomes and block boundaries, while black arrows represent identical break regions shared only by subgenomes of *B. rapa*.

To distinguish the subgenomes in *B. rapa*, syntenic orthologs between *B. rapa* and *A. thaliana* were determined first. We considered two genes between *B. rapa* and *A. thaliana* to be a syntenic ortholog not only by their sequence similarity, but also the number of homologous gene pairs in their flanking chromosomal regions (See Methods). For each *A. thaliana* gene, we counted the number of *B. rapa* syntenic orthologs in *B. rapa*: there were 7,813, 5,439, and 1,675 genes with 1, 2, and 3 syntenic copies in *B. rapa*, respectively. These syntenic orthologs were used as anchors to obtain syntenic genome segments between *B. rapa* and *A. thaliana*. Syntenic segments were then listed along the chromosomes of *A. thaliana*. Like playing jigsaw puzzles, with rules of a) avoiding sequence overlap of the segments' boundaries and b) minimizing the number of inter-chromosomal rearrangement events in *B. rapa*, these listed segments were merged into larger fragments. In each genomic region of *A. thaliana*, three copies of the syntenic fragments were clearly distinguished in *B. rapa* (Figure 2, Table S1 and S2). Using the genomic block numbering in *A. thaliana* [16], we identified the distribution of all the AK blocks in *B. rapa*.

**Figure 3:**
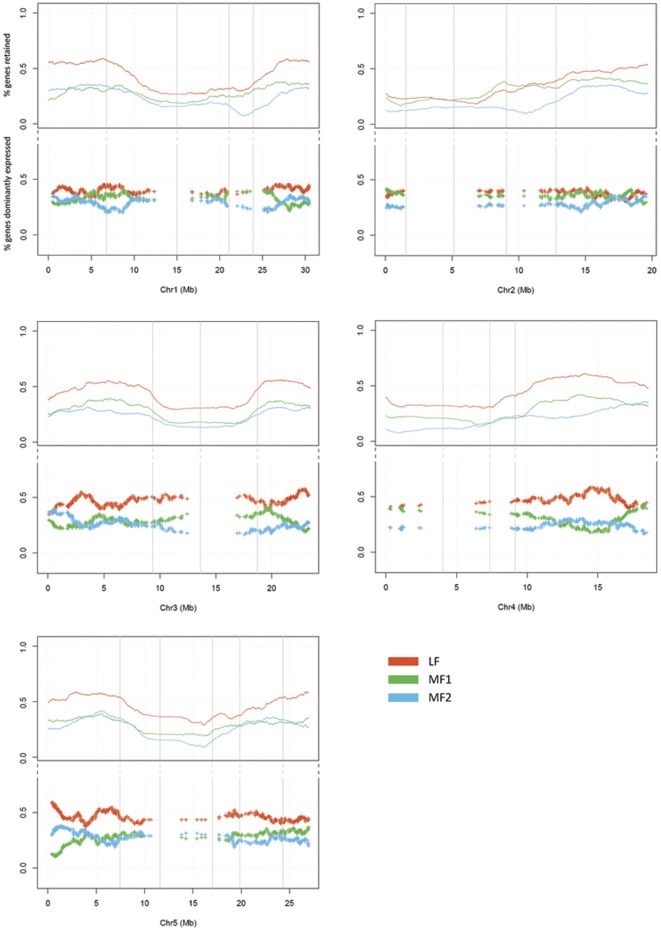
Gene densities and dominance of gene expression in the three subgenomes of *B. rapa* along the chromosomes of *A. thaliana*. The vertical grey lines are the separators of the 24 AK blocks. Red lines and crosses represent subgenome LF, while green and blue ones represent subgenomes MF1 and MF2, respectively. Gene densities are indicated above the lines and dominance of gene expression below. Gene densities were estimated by a window of 1,000 genes with a step of 1 gene moving through the reference genome *A. thaliana*. The ratio of syntenic genes in each of the three *B. rapa* subgenomes for each window was calculated and plotted. For the gene expression, we used a window of 60 genes with a step of 1 gene sliding through the 1,675 *A. thaliana* genes that have three retained syntenic orthologs in *B. rapa*. For each window, we counted the number of genes that have the highest expression among syntenic paralogs for each of the three subgenomes. The expression dominance was then plotted as crossed with the ratio of the highest expressed genes in each subgenome.

When comparing the gene content in the three copies of syntenic fragments, one copy always contained many more genes than the other two; for the other two showing more fractionated copies, they contained slightly different numbers of genes (Figure 3). We further sorted these three copies of syntenic fragments according to their relative gene densities and finally grouped fragments with the highest gene densities as subgenome LF, fragments with moderate gene densities as subgenome MF1, and the fragments with the least genes as subgenome MF2. A full list of the coordinates of these fragments is shown in Supplementary Table S1. As shown in Figure 2, for the three *B. rapa* subgenomes corresponding to chromosomes 1–4 of *A. thaliana*, there are few syntenic fragments with shared identical breakpoints (two for chromosomes 1–3, one for chromosome 4). For *A. thaliana* chromosome 5, the syntenic fragments share six breakpoints. Taking chromosome 1 as an example, one shared breakpoints located between block A-B-C and block D, the other one was between block D and block E.

### Biased gene fractionation in the three subgenomes

We selected 23,716 high-confidence *B. rapa* genes (subsequently referred to as “high confidence genes") by filtering out low confidence genes and genes that have no syntenic orthologs in *A. thaliana* (see Methods). We further selected 1,675 orthologs (5,025 genes) that have syntenic copies in all three subgenomes of *B. rapa*, and referred them as “fully retained homoeologs". These two gene sets were used as basic data in subsequent analyses.

Subgenomes were ordered based on gene densities from high to low and were named as LF, MF1, and MF2. In the 24 AK blocks, gene density in subgenome LF was much higher than in the two MF subgenomes (MF1 and MF2), except for blocks G and H. Subgenome MF1 had slightly, but significantly, more genes than MF2, except for blocks A, B, T and W. This differential gene density was also obvious when we compared them in *A. thaliana* chromosomes that were used as the representatives of AKBr (Table 1 and Figure 3). The observed differentiation of gene density among the three subgenomes indicated that biased gene fractionation exists not only between LF and MFs, but also between MF1 and MF2.

**Table 1:** Biased gene fractionation among the three subgenomes of *B. rapa*, listed along the 24 AK blocks and the chromosomes of *A. thaliana*.

		#Genes			κ^2^ test^a^	
		LF	MF1	MF2	LF & MFs	MF1 & MF2
**Blocks**	**A**	921	498	550	9.94E-18	2.75E-01
	**B**	559	355	356	4.10E-08	9.79E-01
	**C**	299	229	188	1.20E-03	1.76E-01
	**D**	144	117	38	3.11E-04	6.11E-06
	**E**	727	503	356	7.80E-13	4.46E-04
	**F**	1,159	766	638	3.79E-18	1.73E-02
	**G**	8	11	0	8.23E-01	2.67E-02
	**H**	117	198	108	8.59E-02	3.23E-04
	**I**	267	247	56	4.37E-06	5.97E-16
	**J**	783	611	509	7.38E-07	3.44E-02
	**K**	85	76	52	1.91E-01	1.67E-01
	**L**	157	116	74	1.72E-03	3.90E-02
	**M**	192	83	82	5.50E-08	9.56E-01
	**N**	604	386	339	2.13E-10	2.37E-01
	**O**	171	110	51	3.18E-06	1.23E-03
	**P**	95	69	35	4.73E-03	2.45E-02
	**Q**	260	138	132	2.78E-07	8.63E-01
	**R**	1,003	651	623	1.43E-13	6.06E-01
	**S**	190	99	33	1.08E-10	4.74E-05
	**T**	108	28	77	4.10E-04	8.52E-04
	**U**	1,179	770	535	4.52E-24	4.66E-06
	**V**	213	141	141	2.11E-03	9.33E-01
	**W**	433	322	329	1.63E-03	8.90E-01
	**X**	371	202	185	9.13E-10	5.90E-01
	**Unspecified**	652	454	352	3.76E-10	1.26E-02
**Chromosomes**	**Chr1**	2,650	1,790	1,689	2.06E-29	2.35E-01
	**Chr2**	1,477	1,172	838	9.61E-15	1.48E-07
	**Chr3**	2,212	1,435	1,190	1.41E-35	7.87E-04
	**Chr4**	1,658	1,034	767	5.69E-35	9.53E-06
	**Chr5**	2,609	1,606	1,589	1.54E-37	8.51E-01
	**Total**	10,606	7,037	6,073	1.90E-142	2.76E-09

a *: a χ^2^ test was performed between observed gene numbers and expected gene numbers (equal number of genes retained) among subgenomes.*

### Patterns of gene expression among subgenomes LF, MF1, and MF2: genome dominance

Gene expression pattern for all genes in *B. rapa* were measured using mRNA-Seq data. Using Illumina GAII, we generated pair-end reads for mRNA extractions from three organs (root, stem, leaf). Tissues were collected from four-week old Chiifu-401/42 plants grown in a greenhouse. We also used mRNA-seq data from two pooled mRNA extractions of *B. rapa* Chiifu-401/42 and a cultivar line L58CX, respectively (as previously described [13]).

In this analysis, genes from 1,675 fully retained homoeologs were chosen to compare the gene expression patterns of the three subgenomes. Thus, we compared the RNA levels expressed by three syntenic genes in the same sample. We used a two-fold change method to evaluate genes' differential expression, and a gene was considered overexpressed only when it was expressed at least two-fold higher than both of the other two homoeologous genes. When comparing the gene expression dominance between MF1 and MF2, we only took syntenic genes from subgenomes MF1 and MF2 into consideration, and considered a gene to be dominantly expressed if it expressed two-fold higher expression compared to the other copy. In the five expression datasets, we observed that the number of genes from subgenome LF with dominant gene expression was much higher than the number of genes from subgenomes MF1 or MF2 (Table 2, Figure 3). The number of genes from MF1 with dominant gene expression (MFs) was also higher than the number of genes from MF2 (Table 3), although the differences are less obvious compared to those between LF and MFs.

**Table 2:** Dominant gene expression between subgenomes LF and MFs in *B. rapa*.

Organisms	#2-fold changes^a^			Not expressed	Binomial test (LF & MFs)
	LF	MF1	MF2		
**leaf**	393	262	233	106	1.42E-11
**stem**	362	258	228	78	1.48E-08
**root**	356	273	221	75	2.22E-07
**Chiifu**	363	253	216	50	7.06E-10
**L58CX**	355	229	194	29	1.16E-12

a *: number of genes expressed at least two-fold higher compared to both of the other two syntenic genes.*

**Table 3:** Dominant gene expression between subgenomes MF1 and MF2 in *B. rapa*.

Organisms	#2-fold changes		Not expressed	Binomial test
	MF1	MF2		
**leaf**	627	555	165	3.89E-02
**stem**	617	544	121	3.45E-02
**root**	643	537	128	2.22E-03
**Chiifu**	620	538	80	1.73E-02
**L58CX**	616	552	46	6.52E-02

Dominant gene expression was also tested by employing a “horserace experiment" in which a winner can win by any fraction of a reads per kilobase of exon per million reads (RPKM) value, the unit of RNA level. Syntenic gene pairs from the 1,675 fully retained homoeologs and all the possible genome-wide pairs (LF *vs.* MF1 or MF2, MF1 *vs.* LF or MF2, MF2 *vs.* LF or MF1) were tested: genes from the less fractionated subgenome consistently displayed just as great and significant differences to those observed in the two-fold change tests described above (Table S3, S4, S5). We further compared the expression level of all the syntenic gene pairs between subgenomes. The median difference in syntenic gene pairs in which LF expressed at a higher level was marginally higher than the median difference for the pairs in which either MF1 or MF2 expressed at a higher level. The median difference in gene pairs in which MF1 expressed at a higher level was marginally higher than the median difference for gene pairs in which MF2 expressed at a higher level (Table S6).

### Ongoing biased gene fractionation observed in *B. rapa* cultivar lines Turnip and L144

To investigate the recent evolution of the three subgenomes in *B. rapa*, we resequenced a turnip DH (double hybrid) line (a Japanese turnip accession, abbreviated “Turnip") and an inbred line L144 (a rapid cycling oil-like accession) to ∼25 fold of the genome size. For each of these *B. rapa* cultivars, we sequenced three different libraries (insert sizes of 300 bp, 500 bp, and 2000 bp) with pair-end reads to 71 bp. In total we collected ∼80 M and ∼100 M pair-end reads for L144 and Turnip, respectively. Burrows-Wheeler aligner (BWA) and Samtools were used to do the Pair-end reads mapping and variant calling (SNP/InDel) against the reference sequence of released Chiifu genome [22], [23]. Variants covered by at least six unique reads were counted. Both of the two lines were pure homozygous lines; therefore, to guarantee the confidence of the variants, we used only homozygous SNPs/InDels for further analysis (See Methods).

**Figure 4:**
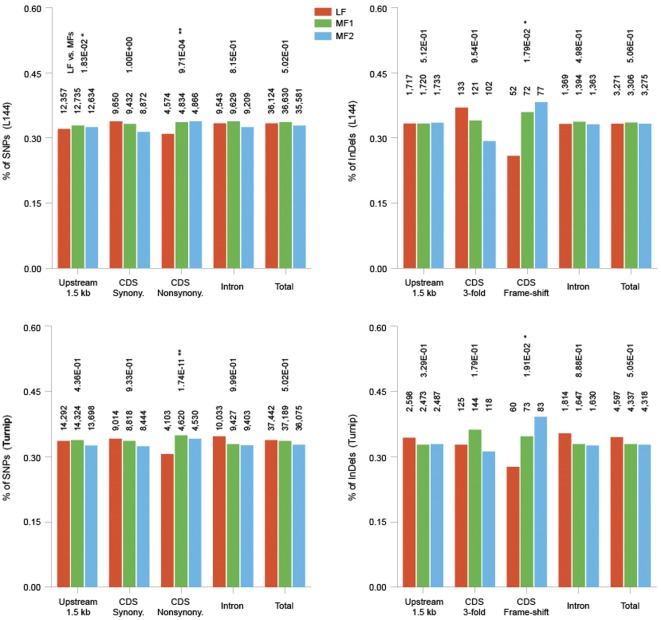
Number of variants (SNPs+InDels) in different regions of fully retained genes (triplets of homeologs) in the three subgenomes of *B. rapa*'s cultivar lines L144 and Turnip. Nonsynonymous SNPs and frameshift InDels in genes from subgenome LF are fewer than those from MFs (p = 9.71E-4, 1.79E-2 for L144; p = 1.74E-11, 1.91E-2 for Turnip). However, no significant differences were observed between MF1 and MF2. Immediately above each bar is the number of variances. Above each group of bars are the *p-values* of the difference in number of variances between LF and MFs produced by the Fisher exact test (single tail).

For all the 23,716 high confidence genes, we found 561,367 SNPs and 45,995 InDels in L144, and 562,935 SNPs, 60,003 InDels in Turnip. The total number of SNPs and InDels in the 1,675 fully retained genes among the three subgenomes was not significantly different from each other in both Turnip and L144 (Table 4), which indicated that the mutations occurred randomly and under equal frequencies in the three subgenomes in the two cultivar lines. For each *B. rapa* gene, were counted the variances located in its 1.5 kb upstream region, exons, and introns, separately. We observed that the genes in subgenome LF always had fewer non-synonymous SNPs and frame-shift InDel mutations than genes in MFs, in both L144 and Turnip (Figure 4). The difference between MF1 and MF2 was not significant, which indicated that subgenome LF was under significantly more selection pressure to sweep the functional mutations in comparison to MFs.

**Table 4:** Variants showing no biased distribution among the three subgenomes in *B. rapa* cultivar lines L144 and Turnip.

Sample	Variant	Subgenomes (per gene)			κ^2^ test
		LF	MF1	MF2	
**L144**	**#SNP**	36,124 (19.86)	36,630 (20.14)	35,581 (19.56)	6.92E-01
	**#InDel**	3,271 (1.80)	3,306 (1.82)	3,275 (1.80)	9.70E-01
**Turnip**	**#SNP**	37,442 (20.58)	37,189 (20.44)	36,075 (19.83)	5.13E-01
	**#InDel**	4,579 (2.53)	4,337 (2.38)	4,318 (2.37)	2.12E-01
**#fully retained homoeologs**		1,675			

## Discussion

The accuracy of subgenome partition is very important for the genome evolution analysis in this study. The relatively recent genome triplication event that occurred in the early stage of *B. rapa* species origin is not as old as that in *C. vinera* (>80 MY) or the most recent tetraploidy in the *A. thaliana* lineage; the AK blocks in the genome of *B. rapa*'s hexaploid ancestor are readily identifiable. Almost all the blocks, although they have been translocated and fused to form the 10 chromosomes of *B. rapa*, are retained intact. All three copies of each block can be separated, facilitating the partitioning of the genome into subgenomes. Using chromosomes of *A. thaliana* as the representative of *B. rapa*'s diploid ancestral karyotype, and employing the rules of 1) nonoverlap of syntenic boundaries and 2) least translocation in *B. rapa*'s chromosomes, we aligned and separated each AK block. This led to a reconstruction of the three subgenomes in *B. rapa*. Most blocks are conserved and are arranged in groups (many blocks existed continuously in all three subgenomes). However, only 11 breakpoints were found to be shared by all three subgenomes, such as the breakpoint located between blocks C and D (Figure 2). These identical breakpoints probably reflect the chromosome boundaries of the real diploid ancestor of *B. rapa*; the *A. thaliana* genome is only a representative of the three true diploid ancestral genomes.

We have proposed a “two-step theory" to explain the *B. rapa* genome evolution [13]. According to this theory, there has been a diploid ancestral genome that contained one copy of the AK blocks. Step 1: two diploid genomes became a tetraploid with two new subgenomes (precursors of MF1 and MF2). Along evolutionary time progressed, loss of genes from the duplicate genomes finally resulted in a fractionated diploid genome (consisting of the two subgenomes MF1+MF2). Step 2: another diploid genome (LF) was added to the fractionated diploid genome, which initiated another round of gene loss. As a result, LF experienced one round of gene loss and retained more genes than MF1 and MF2, which experienced two rounds of gene loss. The two-step theory for genome triplication in *B. rapa* is well supported by the obvious differentiation of gene density between subgenomes LF and MFs. However, we do not exclude the differential methylation hypothesis, because the differences in gene densities could also be maintained by different levels of subgenome methylation [5].

Using mRNA-seq data from both different tissues and pooled tissues of two different accessions of *B. rapa* (Chiifu subspecies *pekinensis* and L58 subspecies *parachinensis*), we found that genes in subgenome LF are dominantly expressed over genes in two MFs (Table 2), and we further found that more genes are dominantly expressed in MF1 compared to MF2 (Table 3). The expression activitys of the three subgenomes was ordered as LF>MF1>MF2. Many studies have noted that methylation represses gene expression [24], [25], [26], [27], [28], thus gene methylation levels might be different among the three subgenomes. Consequently, methylation is likely to have played an important role in *B. rapa* genome evolution.

We observed ongoing biased gene fractionation in *B. rapa* similar to that observed in maize [5]. Using resequencing data of two *B. rapa* accessions (L144, a rapid cycling laboratory accession and Vegetable Turnip VT117, subspecies *rapa*), we found that subgenome LF accumulates significantly fewer non-synonymous SNPs and frameshift InDels than the other two MFs, which correlates with the dominant gene expression in LF compared to MFs. However, this ongoing gene fractionation was a result of the differentiation process of the two subspecies after LF, MF1, and MF2 were combined into one genome. The explanation of biased gene fractionation by genome dominance leaves unanswered questions. In maize, the most likely explanation was proposed to be differential epigenetic marking of subgenomes within an allotetraploid, possibly because allotetraploidy produces epigenetically inherited differentiation of parental genomes [5]. If this is true, the “two-step theory" is consistent with the presence of an allotetraploid during *B. rapa* evolution before LF was added to MFs. We identified many regions in which the gene densities in MF1 are higher than those in MF2 (Table 1). This could also be the result of differential methylation between MF1 and MF2. MF1 and MF2 were merged at the same time; therefore, the different gene density could not be explained by evolution time difference.

Evidence of ongoing biased gene fractionation together with the differential gene expression among the subgenomes of *B. rapa* could be better explained by differential subgenome methylation, although extensive whole genome methylation status data is needed to test this hypothesis. However, this does not exclude the hypothesis of the “two-step theory". Considering the current data, we tend to believe that the “two-step" evolution process, together with methylation differentiation, both played important roles during the evolution of the *B. rapa* genome.

*B. rapa* has an ancient triplicated genome, which is old enough to have fractionated (many genes have been lost after polyploidization), but young enough so that most genes are clearly identifiable in the outgroup, *A. thalian*a. It represents a good model for studying mesopolyploid genome differentiation and offers an opportunity to study evolutionary events on an intermediate timescale. We previously proposed a ‘two-step polyploidization’ hypothesis to explain the gene density difference in subgenomes of *B. rapa* [13]. Here, using more genomic datasets and accurate subgenome partition, we observed dominant expression of genes in a subgenome with higher gene density and ongoing biased gene fractionation between subgenomes of *B. rapa*. We hypothesize that both differential methylation and ‘two-step polyploidization’ played important roles in *B. rapa* genome evolution.

## Methods

### Identification of syntenic genes

For most genes in *B. rapa*, their syntenic genes in *A. thaliana* were determined by both sequence similarity and the colinearity of flanking genes. To obtain syntenic genes, we simplified the analysis of genes that are organized in tandem repeats, using one gene for each array as a representative. Tandem genes were defined as homologous gene clusters that contained no more than one non-homologous gene [20]. All protein sequences derived from predicted gene models of *B. rapa* were then compared to genes of *A. thaliana* using BLASTP. The gene pair with the best hit or any with an e-value<1.0E-20 were further analyzed. In this step, almost every gene of *B. rapa* obtained several homologous genes in *A. thaliana*. The flanking genes of theses homologous gene pairs were then used to exclude nonsyntenic gene pairs. For every *B. rapa* gene, we counted the numbers of best hit gene pairs in *A. thaliana* for both flanking regions of chromosome. From each side, we selected a window of 20 *B. rapa* genes and 100 *A. thaliana* genes and counted the best hit pairs among them as m (left side) and n (right side). We calculated (m+n)/40 as the support ratio. A homologous gene from *A. thaliana* was determined as the syntenic ortholog of a particular *B. rapa* gene when it had the highest flanking support ratio and a ratio >0.4.

### Defining the high confidence homoeolog and fully retained homoeolog gene sets

A *B. rapa* gene was considered as “high confidence" when it was based on start and stop codons and the gene model was supported by cDNA, EST, or mRNA-seq data (40,985 out of the 41,174 total predicted genes satisfied this criterion). Then these high confidence *B rapa* genes were used to find syntenic orthologs in *A. thaliana*; 27,542 of 40,985 genes had syntenic orthologs. Each *A. thaliana* gene had one, two, or three syntenic orthologs in *B. rapa*; multiple orthologs are homoeologs. For each *A. thaliana* gene, if there were any unannotated syntenic blast hits detected in any of the subgenomes in *B. rapa*, then the corresponding homoeologs were eliminated (26,533 of 27,542 genes). Additionally, a homoeolog was removed if it belongs to a local (tandem) gene array in either *B. rapa* or *A. thaliana*, because these genes add complexity to the interpretation of gene expression data and are known to change copy number rapidly (23,716 of 26,533 genes). Finally, 23,716 genes in 14,927 high confidence homoeolog pairs were generated. There were 1,675 high confidence homoeologs comprising three copies in *B. rapa*, one from each of the three subgenomes.

### Gene expression level determination

The mRNA extractions from three organs (root, stem and leaf of Chiifu-401/42 seedlings harvested from 5-leaves plants growing in greenhouse), and two pooled mRNA extractions (*B. rapa* Chiifu-401/42 and a cultivar line L58) [13] were prepared for sequencing. mRNAs were purified by beads with dT-oligos and then fragmented into short sequences using a RNA fragmentation kit (Ambion). cDNA libraries with insert sizes of ∼300 bp were constructed following the manufacturer's instructions (Illumina GAII) and 90 bp paired-end reads were generated using the Illumina HiSeq™ 2000 platform. We obtained about 30∼40 M reads for each of the above RNA samples. Reads containing the sequence adapters or that were of low quality (the number of ‘N’ bases exceeded 5% or the number of bases whose Phred-like score was less than 5 exceeded 50%) were removed from the raw data. Reads were then mapped to genes of Chiifu-401/42 using the SOAP2 package [29], allowing a maximum of two mismatches. Uniquely mapped reads that were located completely in exons were used in the expression level determination. Finally, gene expression levels were calculated in units RPKM [30]. RPKM files for gene expression and the raw reads files are available at http://brassicadb.org/brad/genomeDominanceData.php.

### SNP and InDel discovery in the resequencing data

Pair-ends resequencing data of three libraries, with insert sizes of ∼300 bp, ∼500 bp and ∼2,000 bp for both L144 and Turnip were generated on using an Illumina HiSeq™ 2000 platform. Reads containing the sequence adapters or that were of low quality (the number of ‘N’ bases exceeded 5%, or the average Phred-like score was <20) were removed, and only one copy of the duplicated pair-ends reads was kept. Reads were mapped to the Chiifu genome using BWA [22]. Data from different libraries were independently mapped and the map results were merged to call variants (SNPs and InDels). The Samtools package was used to call variants from the merged file [23]. Only reads with a map quality above 30 and bases with quality above 13 were used to call variants. Heterozygous variants (#reads of reference allele <3, #reads of derived allele >5; “derived allele" means that it is different from the reference allele) were removed, and only homozygous variants that were covered by at least five unique reads were used as data for our experiments.

## Supporting Information

Table S1 **The syntenic segments between chromosomes of** ***A. thaliana*** **and** ***B. rapa*** **.**
(XLS)

Table S2 **High confidence syntenic gene set between** ***A. thaliana*** **and the three subgenomes of** ***B. rapa*** **.**
(XLS)

Table S3 **The number of dominantly expressed genes determined from the fully-retained syntenic paralogs among the three subgenomes of** ***B. rapa*** **by horserace experiment.**
(DOC)

Table S4 **The number of dominantly expressed genes in the three subgenomes determined by horserace experiment from all the pairwise syntenic paralogs in** ***B. rapa*** **.**
(DOC)

Table S5 **The number of dominantly expressed genes in subgenomes MF1 and MF2 determined from the pairwise syntenic paralogs by horserace experiment.**
(DOC)

Table S6 **The median difference of gene expression between all pairwise syntenic paralogs in the three subgenomes in** ***B. rapa*** **.**
(DOC)
