# Supplementary material for: Biased Gene Fractionation and Dominant Gene Expression among the Subgenomes of Brassica rapa
Source: PLoS One. 2012 May 2;7(5):e36442. doi: 10.1371/journal.pone.0036442 (PMC3342247; doi:10.1371/journal.pone.0036442)
Supplement: Table S3 — The number of dominantly expressed genes determined from the fully-retained syntenic paralogs among the three subgenomes of B. rapa by horserace experiment. (DOC) [file pone.0036442.s003.doc]

**Supp. Table S3. The number of dominantly expressed genes determined from the fully-retained syntenic paralogs among the three subgenomes of *B. rapa* by horserace experiment.**

| **Organisms** | **#horserace** | | | **χ^2^ test** |
| --- | --- | --- | --- | --- |
|  | **LF** | **MF1** | **MF2** |  |
| **Leaf** | 669 | 490 | 419 | 5.93E-11 |
| **Stem** | 677 | 489 | 442 | 5.48E-10 |
| **Root** | 666 | 511 | 429 | 1.62E-09 |
| **Chiifu** | 676 | 506 | 446 | 3.68E-09 |
| **L58CX** | 660 | 520 | 466 | 1.31E-06 |
