# Supplementary material for: Biased Gene Fractionation and Dominant Gene Expression among the Subgenomes of Brassica rapa
Source: PLoS One. 2012 May 2;7(5):e36442. doi: 10.1371/journal.pone.0036442 (PMC3342247; doi:10.1371/journal.pone.0036442)
Supplement: Table S4 — The number of dominantly expressed genes in the three subgenomes determined by horserace experiment from all the pairwise syntenic paralogs in B. rapa . (DOC) [file pone.0036442.s004.doc]

**Supp. Table S4.** The number of dominantly expressed genes in the three subgenomes determined by horserace experiment from all the pairwise syntenic paralogs in *B. rapa*.

| **Organisms** | **#horserace ^a^** | | | **χ^2^ test** |
| --- | --- | --- | --- | --- |
|  | **LF** | **MF1** | **MF2** |  |
| **Leaf** | 3,129/6,313 | 2,007/5,138 | 1,569/4,452 | 1.39E-22 |
| **Stem** | 3,139/6,313 | 2,003/5,138 | 1,589/4,452 | 4.34E-22 |
| **Root** | 3,137/6,313 | 1,959/5,138 | 1,531/4,452 | 1.89E-26 |
| **Chiifu** | 3,184/6,313 | 2,008/5,138 | 1,637/4,452 | 1.07E-21 |
| **L58CX** | 3,277/6,313 | 2,040/5,138 | 1,647/4,452 | 8.52E-25 |

*a: digits after ‘/’ denote the number of syntenic gene pairs, digits before ‘/’ denote the number of genes that have the highest expressions in according subgenomes.*
