# Supplementary material for: Biased Gene Fractionation and Dominant Gene Expression among the Subgenomes of Brassica rapa
Source: PLoS One. 2012 May 2;7(5):e36442. doi: 10.1371/journal.pone.0036442 (PMC3342247; doi:10.1371/journal.pone.0036442)
Supplement: Table S5 — The number of dominantly expressed genes in subgenomes MF1 and MF2 determined from the pairwise syntenic paralogs by horserace experiment. (DOC) [file pone.0036442.s005.doc]

**Supp. Table S5.** The number of dominantly expressed genes in subgenomes MF1 and MF2 determined from the pairwise syntenic paralogs by horserace experiment.

| **Organisms** | **#horserace** | | **Binomial test** |
| --- | --- | --- | --- |
|  | **MF1** | **MF2** |  |
| **Leaf** | 1,204 | 1,052 | 1.47E-03 |
| **Stem** | 1,230 | 1,082 | 2.23E-03 |
| **Root** | 1,219 | 1,079 | 3.73E-03 |
| **Chiifu** | 1,221 | 1,130 | 6.34E-02 |
| **L58CX** | 1,253 | 1,161 | 6.40E-02 |
