# Supplementary material for: Biased Gene Fractionation and Dominant Gene Expression among the Subgenomes of Brassica rapa
Source: PLoS One. 2012 May 2;7(5):e36442. doi: 10.1371/journal.pone.0036442 (PMC3342247; doi:10.1371/journal.pone.0036442)
Supplement: Table S6 — The median difference of gene expression between all pairwise syntenic paralogs in the three subgenomes in B. rapa . (DOC) [file pone.0036442.s006.doc]

**Supp. Table S6.** The median difference of gene expression between all pairwise syntenic paralogs in the three subgenomes in *B. rapa*.

| **Organisms** | **LF expressed higher** | | **MF1 expressed higher** | | **MF2 expressed higher** | |
| --- | --- | --- | --- | --- | --- | --- |
|  | **LF/MF1** | **LF/MF2** | **MF1/LF** | **MF1/MF2** | **MF2/LF** | **MF2/MF1** |
| **Leaf** | 3.15 | 3.35 | 2.70 | 3.26 | 2.48 | 2.72 |
| **Stem** | 2.96 | 3.35 | 2.54 | 2.95 | 2.37 | 2.61 |
| **Root** | 3.41 | 4.03 | 2.85 | 3.52 | 2.67 | 2.79 |
| **Chiifu** | 2.89 | 3.32 | 2.74 | 2.84 | 2.43 | 2.71 |
| **L58CX** | 3.05 | 3.03 | 2.74 | 3.12 | 2.69 | 2.91 |
